# The natural compound forskolin synergizes with dexamethasone to induce cell death in myeloma cells via BIM

**DOI:** 10.1038/srep13001

**Published:** 2015-08-26

**Authors:** Virginie Follin-Arbelet, Kristine Misund, Elin Hallan Naderi, Hege Ugland, Anders Sundan, Heidi Kiil Blomhoff

**Affiliations:** 1 Department of Molecular Medicine, Institute of Basic Medical Sciences, University of Oslo, PO Box 1112-Blindern, N-0317 Oslo, Norway; 2 KG Jebsen Center for Myeloma Research and Department of Cancer Research and Molecular Medicine, Norwegian University of Science and Technology, N-7489 Trondheim, Norway

## Abstract

We have previously demonstrated that activation of the cyclic adenosine monophosphate (cAMP) pathway kills multiple myeloma (MM) cells both *in vitro* and *in vivo*. In the present study we have investigated the potential of enhancing the killing of MM cell lines and primary MM cells by combining the cAMP-elevating compound forskolin with the commonly used MM therapeutic drugs melphalan, cyclophosphamide, doxorubicin, bortezomib and dexamethasone. We observed that forskolin potentiated the killing induced by all the tested agents as compared to treatment with the single agents alone. In particular, forskolin had a synergistic effect on the dexamethasone-responsive cell lines H929 and OM-2. By knocking down the proapoptotic BCL-2 family member BIM, we proved this protein to be involved in the synergistic induction of apoptosis by dexamethasone and forskolin. The ability of forskolin to maintain the killing of MM cells even at lower concentrations of the conventional agents suggests that forskolin may be used to diminish treatment-associated side effects. Our findings support a potential role of forskolin in combination with current conventional agents in the treatment of MM.

Multiple myeloma (MM) is an incurable plasma cell malignancy that accounts for approximately 1% of neoplastic disorders worldwide^1^. It is characterized by accumulation of malignant cells in the bone marrow leading to osteolytic bone lesions and impaired hematopoiesis. Clinical manifestations include anemia, recurrent infections, bone pain, fractures and hypercalcemia^2^. Patients with MM have a median survival of 3 to 5 years^3^. The standard treatment of the disease is chemotherapy with or without autologous stem cell transplantation^3^. Although the emergence of new drugs has changed the landscape of MM treatment, patients experience drug-mediated side effects such as fatigue, anemia, and peripheral neuropathy^4^,^5^,^6^. Furthermore, in almost all cases, patients relapse and are resistant to subsequent therapy^7^. Consequently, new therapeutic strategies are required to improve the outcome and the survival of MM patients.

Modulation of the cAMP signaling has previously shown promising results in MM^8^,^9^. We demonstrated that elevation of intracellular cAMP levels induces apoptosis in both MM cell lines and patient tumor cells^8^,^10^. Additionally, we observed that injection of the cAMP-elevating agent forskolin delays MM tumor growth in a mouse model. Importantly, interleukin 6 (IL-6), a central cytokine promoting MM cells growth and survival^11^,^12^, did not prevent the cAMP-mediated apoptosis of the MM cells^10^. Taken together with the reported synergy between adenosine receptor agonists and phosphodiesterase inhibitors to inhibit cell proliferation and induce death of MM cells^13^, our previous observations^8^,^10^ indicate that cAMP-elevating agents such as forskolin might have a therapeutic potential in MM.

Forskolin is a diterpene produced by the roots of the Indian plant *Coleus forskohlii*^14^. This plant has been used for centuries in Hindu Ayurvedic medicine^15^. Forskolin raises intracellular cAMP levels by directly activating adenylyl cyclase, an enzyme that generates cAMP from ATP. Although forskolin has been reported to modulate other cellular processes, such as ion channels^16^,^17^, our previous studies have demonstrated that it induces MM cell death solely through elevation of cAMP levels^8^,^10^. Forskolin or its derivative, colforsin daropate, has been tested in several clinical settings including cardiovascular diseases^18^,^19^, obesity^20^, psychiatric disorders^21^ and asthma^22^, and clinical studies with oral administration of forskolin 50 mg/day have not revealed significant side effects in patients^20^,^23^. In the present study, we have investigated the effect of forskolin in MM cells in combination with clinically relevant MM therapeutic agents: DNA-damaging agents including the anthracycline antibiotic doxorubicin, the alkylating agent cyclophosphamide and melphalan, the proteasome inhibitor bortezomib, and the glucocorticoid dexamethasone. Cell death was evaluated in multiple myeloma cell lines as well as in primary MM cells from patients.

We here show that forskolin together with most of the agents tested has an additive effect on cell death. Furthermore, even suboptimal concentrations of forskolin alone gives as good, or even better, response as any of the single therapeutic drug. In particular, dexamethasone and forskolin had a synergistic effect on cell death in H929 and OPM-2 cells. We found that the cell death induced by the combination of dexamethasone and forskolin involved the induction of the pro-apoptotic protein BIM. Taken into account the adverse side effects of current MM therapeutic agents, we propose forskolin as an adjuvant in combination with dexamethasone to reduce the side effects and increase the efficiency of MM treatments.

## Materials and Methods

### Chemicals and Antibodies

Forskolin and dexamethasone (Sigma-Aldrich, St Louis, MO, USA) were dissolved in dimethyl sulfoxide (DMSO) at a concentration of 30 mM and 10 mM respectively. Stock solutions were aliquoted and stored at −20 °C. Melphalan (Sigma-Aldrich) was dissolved in acidified ethanol at a concentration of 50 mM, and aliquots were stored at −20 °C. 4-hydro-peroxy-cyclophosphamide (Niomech, Bielefeld, Germany) and doxorubicin (Sigma-Aldrich) were dissolved in sterile distilled water at a concentration of 10 mM, and aliquots were stored at −80 °C or 4 °C respectively. Cyclophosphamide monohydrate (Sigma-aldrich) was dissolved in sterile distilled water to 40 mg/ml. Bortezomib (Selleck Chemicals LLC, Houston, TX, USA) was dissolved in DMSO at a concentration of 10 mM, and aliquots were stored at −80 °C. Propidium iodide (PI), DMSO, and bovine serum albumin (BSA) were purchased from Sigma-Aldrich.

Antibodies against caspase 3, BIM, ERK1/2, phosphor-ERK1/2 (Thr202/Tyr204), and PARP were purchased from Cell Signaling Technologies (Danvers, MA, USA). Antibody against alpha tubulin (Novus Biologicals, Littleton, CO, USA) was used as loading control. Anti-rabbit and anti-mouse HRP-conjugated secondary antibodies were purchased from Bio-Rad (Hercules, CA, USA).

### Cell Lines and Cell Culture

The human myeloma cell lines (HMCLs) U266, NCI-H929, RPMI 8226 and OPM-2 were purchased from the American Type Culture Collection (ATCC, Manassas, VA, USA). U266, RPMI 8226 and OPM-2 cells were cultured in RPMI 1640 (Lonza, Verviers, Belgium) containing 2 mM L-glutamine, supplemented with 15% (for U266 cells) or 10% (for OPM-2 and RPMI 8226 cells) heat-inactivated fetal bovine serum (FBS) (Sigma-Aldrich), 100 U/ml penicillin and 100 μg/ml streptomycin (Life Technologies, Grand Island, NY, USA). H929 cells were cultured in RPMI 1640 (Life Technologies) containing 2 mM L-glutamine, supplemented with 15% heat-inactivated fetal bovine serum (FBS) (Sigma-Aldrich), 0.05 mM 2-mercaptoethanol (Sigma-Aldrich), 1 mM sodium pyruvate (Sigma-Aldrich), 100 U/ml penicillin and 100 μg/ml streptomycin (Life Technologies). The human multiple myeloma INA-6 cell line was a kind gift from Dr. M. Gramatzki (Erlangen, Germany) and was cultured in RPMI 1640 (Life Technologies) containing 2 mM L-glutamine, supplemented with 1 ng/ml IL-6 (Life Technologies), 12 μg/ml gentamycin (Sigma-Aldrich) and 10% heat-inactivated FBS (Lonza).

### Primary Patients Samples

To obtain primary myeloma cells, CD138+ cells were isolated from bone marrow specimens obtained through the Norwegian Myeloma Biobank using RoboSep automated cell separator and Human CD138 Positive Selection Kit (StemCell Technologies). All subjects provided informed consent to participate in the study. This study was carried out in accordance with the approved guidelines by the Regional Ethics Committee.

Bone marrow stromal cells (BMSCs) were prepared as described previously^24^. BMSC (2500 cells/well) were seeded in 96-well plates and allowed to adhere for at least 3 hours before addition of primary myeloma cells (5 × 10^3^ cells/well). The cells were cultivated in RPMI medium supplemented with 2% human serum in a total volume of 200 μL/well, and drugs were added at the concentrations indicated. All samples were run in duplicate.

### Small interference RNA transfections

Control siGenome Non-Targeting pool siRNA (D-001206-14) and BIM small interfering RNA (siRNA) (D-121190-01, D-121190-02, D-121190-03 and D-121190-04) were obtained from Thermo Fisher Scientific Biosciences GmbH (St. Leon-Rot, Germany). OPM-2 cells were transfected with siRNA using Lipofectamine® RNAiMAX Transfection Reagent (Life technologies) according to manufacturer’s protocol. Cells were rested 72 hours before being plated in experimental conditions. H929 cells were transfected with siRNA using Amaxa nucleofection technology (Lonza). Briefly, 2 × 10^6^ cells were resuspended in Ingenio^TM^ Electroporation Solution (Mirus Bio LLC, Madisson, WI, USA) and electroporated using program T-16. Cells were rested for 5 hours before being plated in experimental conditions. Knockdown was confirmed by western blot.

Specific cell death was calculated using the following equation: % specific cell death = (% experimental cell death in the drug-treated sample - % spontaneous cell death in the absence drug)/(100 - % spontaneous cell death in the absence of drug) x 100.

### Analysis of Cell Death by Flow Cytometry

Flow cytometry analysis was performed on a FACSCalibur (BD Biosciences). For determination of cell viability by exclusion of PI, cells in culture were incubated with PI (20 μg/ml) prior to analysis. Percentage of dead cells was determined by gating for PI positive cells^25^,^26^.

### ScanR Viability Assay

This assay was performed as described previously^24^. Briefly, after 3 days of incubation, YO-PRO-1 (1 μM) (Life Technologies) was added to the wells, and the plates incubated for 30 min at 37 °C. DRAQ5 (2,5 μM) (eBioscience, San Diego, CA, USA) was added to the wells 15 min before image acquisition without further processing. The ScanR microscope-based screening platform was used for automated image acquisition (Olympus). Images were analyzed using the Olympus ScanR Analysis software, where the cells were classified and gated by size, morphology and fluorescence intensity. Single, circular cells were classified and gated as myeloma cells. YO-PRO-1 is a DNA-binding dye that stains apoptotic cells. Myeloma cells that showed low YO-PRO-1 staining and high intensity DRAQ5 staining were characterized as viable.

### Immunoblot Analysis

Cells were lysed in RIPA buffer (50 mM Tris [pH7.5], 150 mM NaCl, 1% NP-40, 0.1% SDS, 0.5 mM EDTA, 50 mM NaF, 10 mM β-glycerophosphate, 1 mM Na_3_VO_4_, 0.2 mM phenylmethylsulfonyl fluoride [PMSF], 10 μg/ml leupeptin, and 0.5% aprotinin) and equal amounts of proteins were separated by SDS-PAGE (Bio-Rad). After transfer to a nitrocellulose membrane (GE Healthcare, Amersham, UK) using a semi-dry transfer cell (Bio-Rad), proteins were detected by standard immunoblotting procedures. In brief, the nitrocellulose membranes were washed in Tris buffered saline and 0.1% Tween (TBST) and incubated in blocking solution (5% non-fat dry milk in TBST) at room temperature. After washing, the membranes were incubated overnight at 4 °C with primary antibodies diluted in 5% non-fat dry milk in TBST or 5% BSA in TBST. After washing in TBST, the membranes were incubated for 1 hour with HRP-conjugated secondary antibody diluted in blocking solution, followed by a final washing at room temperature. Immunoreactive proteins were visualized with the enhanced chemiluminescence detection system SuperSignal® West Dura Extended Duration substrate (Thermo Scientific, Rockford, IL, USA) according to the manufacturer’s protocol. Images were acquired with a Syngene ChemiGenius camera and presented by the GeneSnap software tool (Syngene, Cambridge, UK); the resulting SGD files were converted to JPEG files for image presentation. Densitometric analyses were performed on the primary SGD files to preserve maximal information, using the GeneTools 4.01 program (Syngene).

### Statistical Analysis

The paired-samples t-test and the Wilcoxon matched-pairs signed rank test were applied to check the significance in cell lines experiments and primary patients’ samples respectively using the Graph Pad Prism 6 software (san Diego, CA, UAS). For cell lines experiments, histograms show mean values of the indicated number of experiments, with error bars corresponding to SEM values.

### Determination of synergism by the combination index (CI) method

The interaction of forskolin and dexamethasone was determined by combination index (CI) method^27^ using the CompuSyn software program (ComboSyn Inc., Paramus, NJ, USA) to determine whether the combination was antagonistic, additive or synergistic. Data from cell viability by exclusion of PI were expressed as the fractional inhibition by the individual drugs or the combination in drug-treated cells, and CI values between two drugs were generated. CI values greater than 1 indicate antagonism, CI value of 1 indicates an additive effect, and CI values of less than 1 indicate synergy.

## Results

### Forskolin in combination with therapeutic agents: effects on death of multiple myeloma cell lines

**Figure 1:**
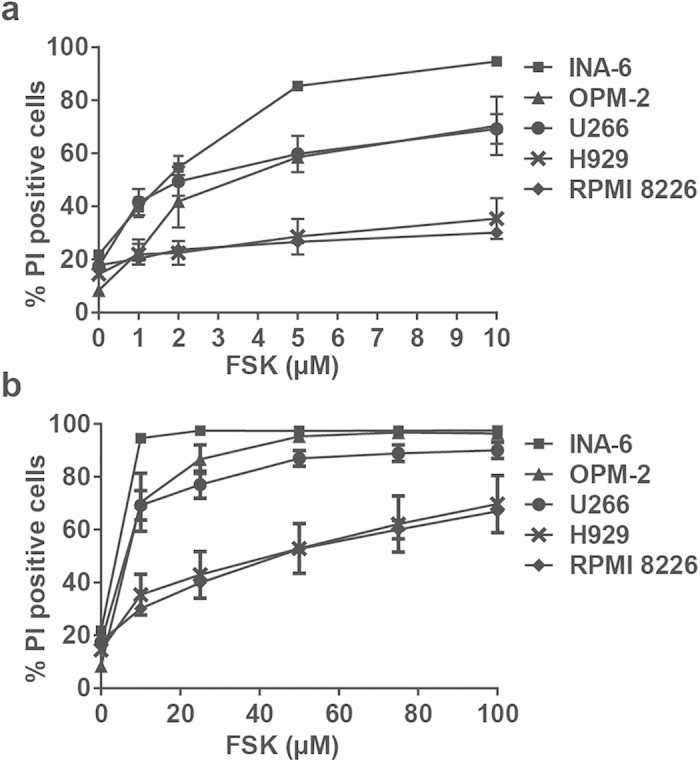
Dose dependent cell death induced by forskolin in five different HMCLs. U266, H929, INA-6, RPMI 8226 and OPM-2 cells were treated for 72 hours with low (panel **a**) and high (panel **b**) concentrations of forskolin (FSK). Cell death was assessed by propidium iodide exclusion (PI). The results are presented as mean of at least three independent experiments ± SEM.

In order to determine the effects of forskolin and the different therapeutic agents on death of human myeloma cell lines (HMCLs), incorporation of PI was measured after 72 hours of treatment. As shown in Fig. 1, forskolin alone induced cell death in a dose-dependent manner in the five HMCLs tested, with U266, OPM-2 and INA-6 being more sensitive than H929 and RPMI 8226 cells. Hence, in the highly forskolin-sensitive cell line U266, forskolin more than doubled the extent of basal cell death at a concentration of 1 μM in U266 or 5 μM in H929, whereas the percentage of dead cells more than tripled when forskolin was used at a concentration of 5 μM in U266 and 50 μM in H929.

**Figure 2:**
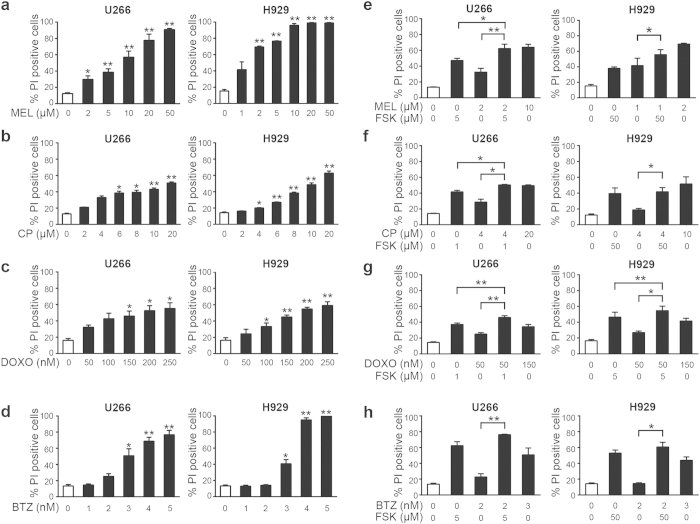
Effect of melphalan, 4-hydro-peroxy-cyclophosphamide, doxorubicin, and bortezomib alone or in combination with forskolin on cell death in U266 and H929 cells. U266 and H929 cells were treated with the indicated doses of melphalan (MEL) alone (panel **a**) or in combination with forskolin (FSK) (panel **e**), 4-hydro-peroxy-cyclophosphamide (CP) alone (panel **b**) or in combination with FSK (panel **f**), doxorubicin (DOXO) alone (panel **c**) or in combination with FSK (panel **g**), bortezomib (BTZ) alone (panel **d**) or in combination with FSK (panel **h**). Cell death was assessed by PI exclusion after 72 hours of treatment. The histograms represent the mean of at least three independent experiments ± SEM. *p < 0.05, **p < 0.01.

We next evaluate the benefit of combining forskolin with different agents that are all frequently utilized in treatment of MM^28^. First, we assessed the death of U266 and H929 cells treated with selected concentration of the DNA damaging agents melphalan, cyclophosphamide and doxorubicin as well as to the proteasome inhibitor bortezomib using ranges that are commonly applied in preclinical models^29^,^30^,^31^,^32^. Both cell lines responded to the different drugs *per se* with variable sensitivity (Fig. 2a–d). Notably, only 2 μM melphalan was required to obtain 70% cell death in H929 cells, whereas a ten times higher concentration of melphalan was needed to reach the same level of cell death in U266 (Fig. 2a). We next combined forskolin with these agents using a suboptimal concentration of forskolin together with a low dose of the particular agent. The level of cell death obtained by these treatments was compared to a single higher dose of that same agent. The rationale behind these experiments was to explore the possibility that the combined treatment with forskolin would make it possible to lower the concentrations of the therapeutic agents and still obtain a beneficial level of cell death. If so, this would potentially reduce the side effects of the conventional agents in a clinical setting. In U266 cells, 5 μM of forskolin significantly increased the level of melphalan-induced cell death from approximately 30% (in the presence of 2 μM melphalan alone) to 60% (when forskolin was combined with 2 μM of melphalan) (Fig. 2e, left panel). Notably, the combination of melphalan (2 μM) and forskolin (5 μM) enhanced the cell death to the same extent as a single high dose (10 μM) of melphalan alone. Forskolin also significantly improved the cell death induced by a single lower dose of melphalan in H929 cells (Fig. 2e, right panel), but in these cells a higher concentration (50 μM) of forskolin was required. An even lower concentration of forskolin (1 μM) was sufficient to enhance the death of U266 cells induced by 4 μM of cyclophosphamide from 30% to 50% (Fig. 2f, left panel). Again the combined treatment with forskolin induced the same level of cell death as a five times higher concentration of cyclophosphamide alone. Comparable results were obtained upon treatment with doxorubicin (Fig. 2g). Hence, in both cell lines, forskolin significantly enhanced the cell death induced by 50 nM of doxorubicin (from 25% to 45%), and the combination with forskolin induced the same level of cell death as the three times higher concentration of doxorubicin alone (Fig. 2g). Forskolin also significantly improved bortezomib-induced cell death in both cell lines (Fig. 2h). It is noteworthy that in most of the cases we presently have analyzed, even a low concentration of forskolin alone was nearly as effective as the combination with a low concentration of the therapeutic agent. The exceptions, i.e. where there was a statistical significant higher cell death obtained by combining forskolin with a given agent as compared to forskolin alone, are indicated by asterisks in Fig. 2.

### The combination of forskolin and dexamethasone synergistically enhanced the death of HMCLs.

**Figure 3:**
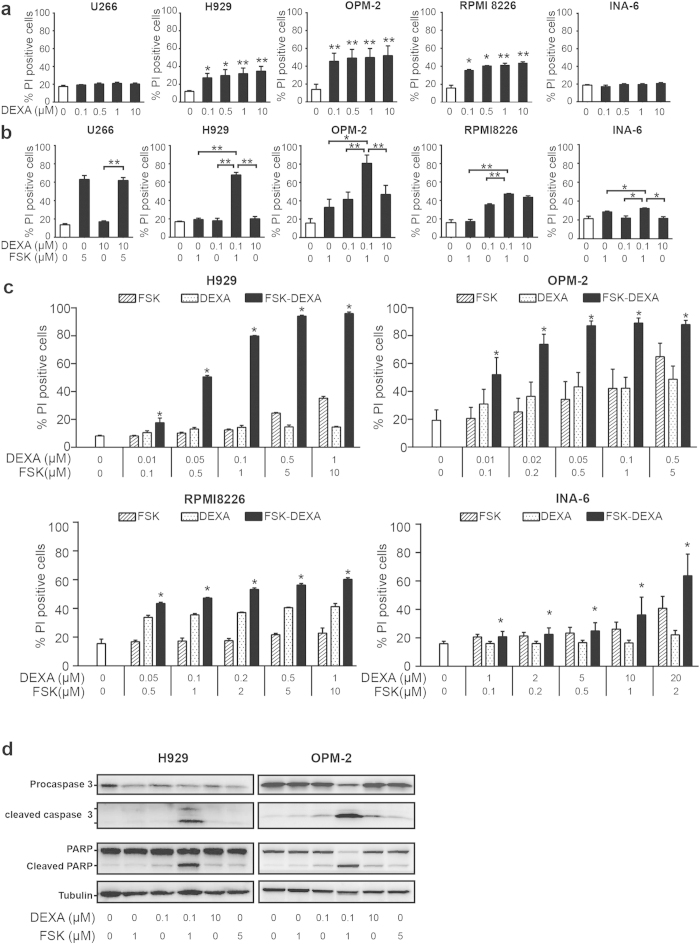
Combination of low doses of forskolin and dexamethasone induces synergistic cell death in HMCLs. (Panels **a** and **b**): U266, H929, OPM-2, RPMI8226 and INA-6 cells were treated for 72 h with the indicated doses of dexamethasone (DEXA) (panel **a**) or with the indicated concentrations of forskolin (FSK) alone or in combination with DEXA (panel **b**). Cell death was assessed by propidium iodide exclusion (PI). The histograms represent the mean of at least three independent experiments ± SEM. *p < 0.05, **p < 0.01 relative to untreated cells. (Panel **c**): H929, OPM-2, RPMI8226 and INA-6 cells were treated with the indicated concentrations of FSK or DEXA alone or in combination for 72 h. Cell death was assessed by propidium iodide exclusion (PI). Histograms represent average of at least three independent experiments ± SEM. *combination is synergistic (CI > 1). Panel d: H929 and OPM-2 were treated with the indicated doses of DEXA and FSK. After 24 h, cells were subjected to western blot analysis for full length and cleaved products of caspase 3 and PARP. Tubulin was used as a loading control. One of three representative experiments is shown. The full size blots can be found in the Supplementary Figure S1.

Unlike bortezomib and the different DNA damaging agents tested, the glucocorticoid dexamethasone alone had no or modest effect in U266 and H929 respectively (Fig. 3a). The OPM-2 and the RPMI8226 MM cell line, however, were sensitive to dexamethasone treatment (Fig. 3a). Remarkably, dexamethasone was the only agent that was found to induce strong synergy at a low concentration of forskolin. Hence, in H929 cells, 1 μM of forskolin and 0.1 μM of dexamethasone alone did not induce any cell death, whereas the combination between these two compounds strongly enhanced the cell death from approximately 20% to 70% (Fig. 3b). The same combination also enhanced cell death in OPM-2 cells as compared to single agents alone (Fig. 3b). More moderate effect was obtained in RPMI8226 and INA-6 cell lines. Dexamethasone had no effect alone or in combination with forskolin in U266 cells (Fig. 3b). The combinatorial effect of forskolin with dexamethasone was evaluated by the CI method, and synergy was observed across a wide range of concentrations for the four MM cell lines tested (Fig. 3c). However, it is noteworthy that not all cell lines responded to the same extent. Hence, synergistic killing was stronger in H929 and OPM-2 cells as compared to the killing of RPMI8226 and INA-6 cells.

In order to establish that apoptosis was the mode of cell death induced by forskolin in combination with dexamethasone, apoptosis in terms of caspase activation was assessed in H929 and OPM-2 cells. Hence, caspase activation was determined by western blot analysis of cleaved caspase 3 and the caspase substrate poly (ADP-Ribose) polymerase (PARP). In accordance with the results on PI-incorporation, the western blot analysis showed that forskolin synergized with dexamethasone also in terms of caspase activation (Fig. 3d). Hence, the combination of 1 μM of forskolin and 0.1 μM of dexamethasone strongly enhanced the cleavage of both caspase 3 and PARP, whereas each of the compounds alone had negligible effects.

### Knock down of BIM partially prevents apoptosis induced by the combination of dexamethasone and forskolin

**Figure 4:**
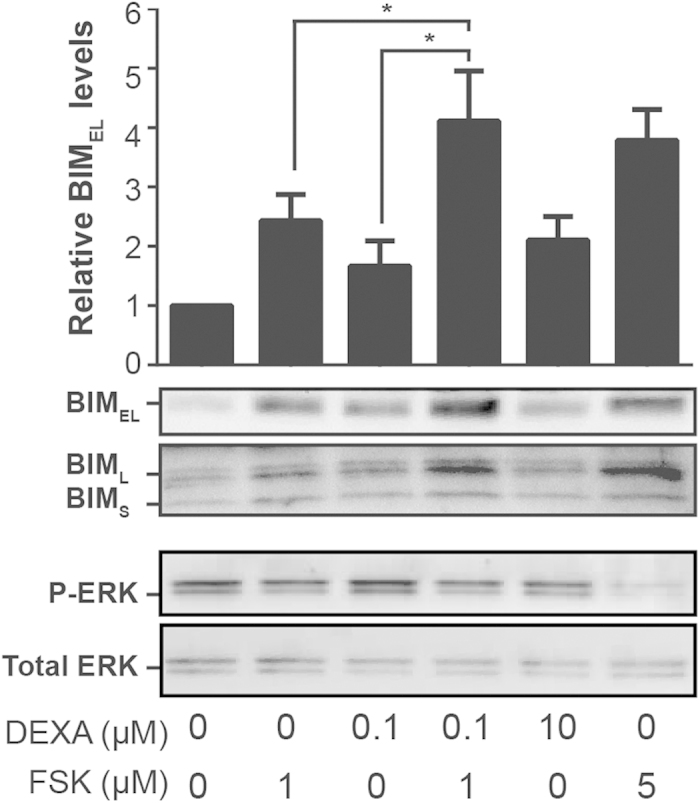
Combination of low doses of forskolin and dexamethasone enhances the expression of BIM. H929 cells were incubated with the indicated concentrations of forskolin (FSK) and dexamethasone (DEXA). After 4 hours, cells were collected and subjected to western blot analysis of BIM and tubulin. The full size blots can be found in the Supplementary Figure S2. The histograms show the levels of BIM_EL_ relative to the level in untreated cells set as 1. The results are presented as mean ± SEM of four independent experiments. *p < 0.05.

BCL-2 family members are essentials players of the apoptotic machinery. Previous studies have established a role of the BH3-only Bcl-2 family member BIM in the cAMP-promoted apoptosis^33^,^34^. In addition, BIM has been suggested to play an important role in glucocorticoid-mediated apoptosis^35^. There are three splice variants of BIM protein (BIM_EL_, BIM_L_ and BIM_S_) and all of them are pro-apoptotic. We therefore assessed the expression of all 3 isoforms of BIM after dexamethasone and forskolin treatment in H929 cells. Interestingly, treatment of H929 cells with combined low doses of forskolin and dexamethasone triggered a four time increase in BIM_EL_ expression levels compared to control (Fig. 4), and the combination was significantly more potent to increase BIM_EL_ levels than dexamethasone or forskolin alone. BIM_L_ and BIM_S_ were also strongly enhanced by forskolin plus dexamethasone treatment. In contrast, examination of the other BCL-2 family members BCL-2, BCL-XL, and BAX revealed only minor alterations in protein expression (data not shown). ERK1/2 has been shown to promote BIM degradation^36^. Consistently, we observed that forskolin alone or in combination with dexamethasone inhibited the activating phosphorylation of ERK (Fig. 4), suggesting that BIM upregulation could result from inhibition of ERK signaling.

**Figure 5:**
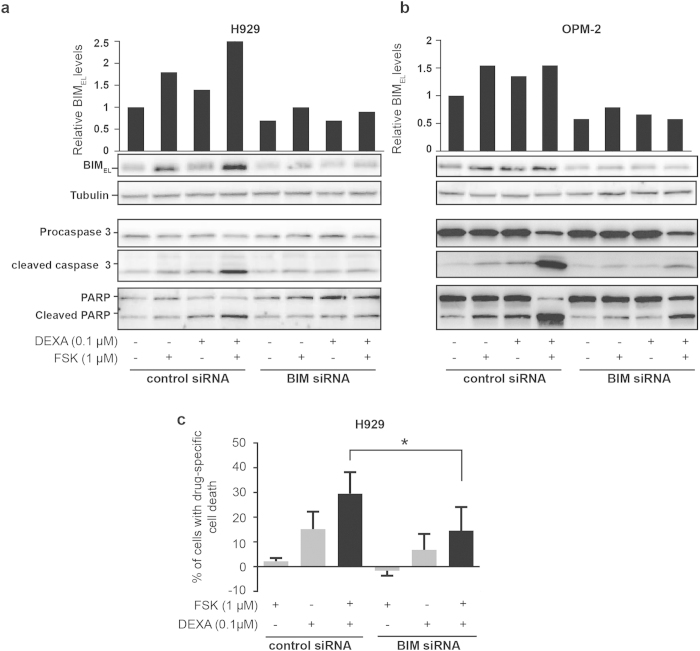
Knock down of BIM partially prevents apoptosis induced by the combination of dexamethasone and forskolin. H929 and OPM-2 cells were transfected with control siRNA or BIM siRNA using the Amaxa nucleofection technology and Lipofectamine transfection reagent, respectively. Forskolin (FSK, at a final concentration of 1 μM) alone or in combination with dexamethasone (DEXA, at a final concentration of 0.1 μM) was added to the cells 5 hours (for H929 cells) or 72 hours (for OPM-2 cells) after transfection. After 4 hours of treatment, the H929 cells (panel **a**) and OPM-2 cells (panel **b**) were collected and subjected to western blot analysis of BIM and tubulin, whereas the levels of full length and cleaved products of caspase 3 and PARP were assessed after 24 h of treatment. The full size blots can be found in the Supplementary Figure S3. (Panel **c**): After 48 hours of treatment, cell death was assessed by PI exclusion in U266. Histograms represent the percentage of drug-specific cell death of 3 independent experiments. *p < 0.05.

To determine whether BIM mediated the apoptosis induced by the combination of forskolin and dexamethasone, BIM expression was knocked down by using the siRNA strategy. Quantification of protein bands by densitometry revealed that knock down reduced the levels of BIM_EL_ by 40% in OPM-2 cells and 30% in H929 cells as compared with control siRNA, and the levels of BIM_L_ and BIM_S_ were similarly knocked down (data not shown). Importantly, the knock down of BIM inhibited the apoptotic cell death induced by the combination of forskolin and dexamethasone in both H929 and OPM-2 MM cells. Hence, the induced activation of caspases, as envisioned by proteolytic cleavage of PARP and caspase 3, was markedly inhibited upon knock down of BIM both in H929 cells (Fig. 5a) and to a greater extent in OPM-2 (Fig. 5b). Furthermore, the cell death induced by the combination of forskolin and dexamethasone was significantly reduced by knocking down BIM in H929 cells as compared with cells treated with control siRNA (Fig. 5c). Knocking down BIM also decreased the cell death induced by combining forskolin and dexamethasone in OPM-2 cells, but the effect was less pronounced (data not shown). Taken together, these results suggest that apoptosis induced by the combination of forskolin and dexamethasone is at least in part mediated via BIM.

### Forskolin enhances cell death mediated by therapeutic agents on primary cells from multiple myeloma patients

**Figure 6:**
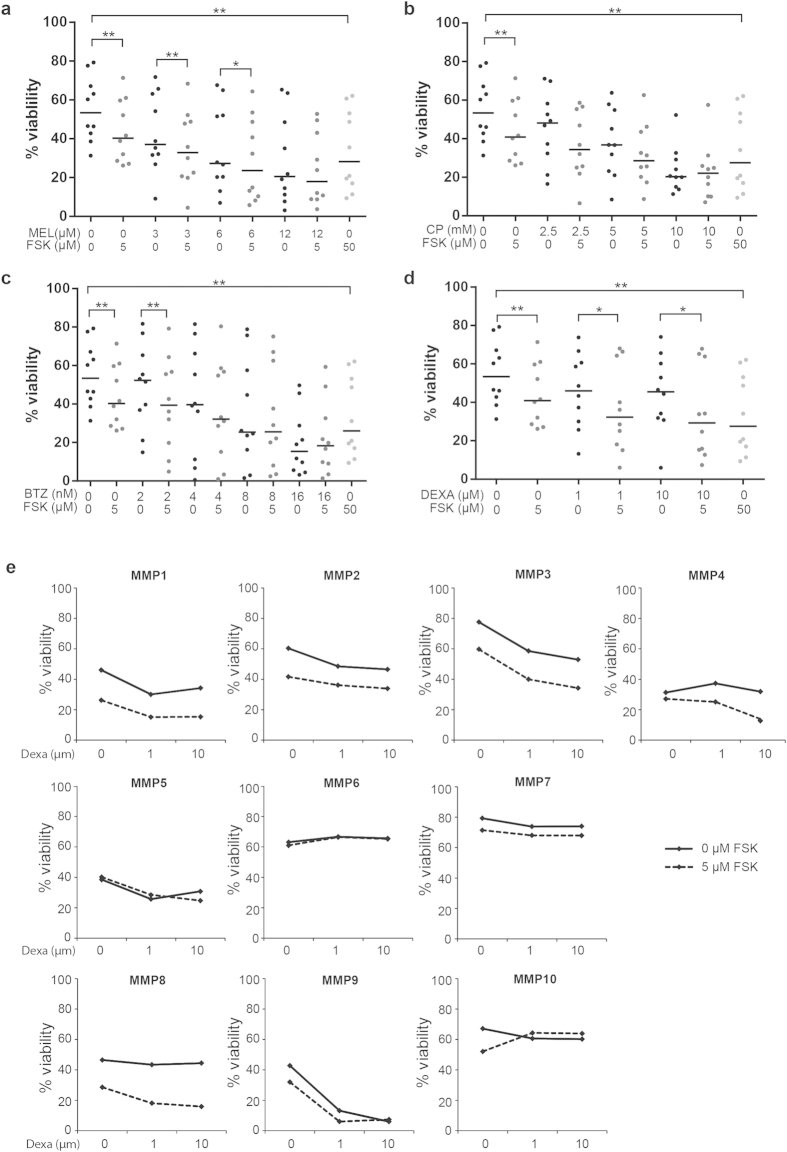
Effect of forskolin in combination with conventional therapeutic agents on primary MM cells. CD138+ B cells isolated from ten multiple myeloma patients (MMP) were co-cultured with bone marrow stromal cells (BMSCs) for 72 h with or without the indicated dose of forskolin in combination with the indicated concentrations of melphalan (panel **a**), cyclophosphamide monohydrate (panel **b**), bortezomib (panel **c**) or dexamethasone (panel **d**). Cells were stained with DRAQ5/YO-PRO-1, and cell viability was evaluated by using the ScanR microscope as previously described^24^. *P < 0.05, **P < 0.01. Panel e: results from panel d presented as bar lines for each patients.

To support the notion that forskolin can be of potential use in MM therapy, CD138+ B cells isolated from ten patients with MM were co-cultured with bone marrow stromal cells and treated with forskolin in combination with melphalan, cyclophosphamide, bortezomib or dexamethasone, respectively. The cell death of primary myeloma cells was assessed after 72 h hours of treatment by using an automated fluorescence microscope platform as previously described^24^. In accordance with the results obtained on cell lines (Figs 2 and 3), forskolin increased the activity of melphalan (Fig. 6a), bortezomib (Fig. 6c) and dexamethasone (Fig. 6d). The effect was statistically significant with low concentrations of bortezomib and melphalan, and with the two concentrations of dexamethasone that were tested. Forskolin also increased the activity of cyclophosphamide, however without reaching statistical significance (Fig. 6b). It is noteworthy that the combination of forskolin and dexamethasone was the most efficient treatment combination in terms of killing the primary MM cells. Hence, 5 μM of forskolin in combination with 1 μM of dexamethasone reduced the viability of the MM cells to a larger extent than even a ten times higher concentration of dexamethasone alone (Fig. 6d). It should be noted that not all patient-derived MM cells responded to the combination of forskolin and dexamethasone. Hence, forskolin had no effect on dexamethasone-mediated killing of the three patient-derived cells MMP5, MMP6 and MMP10, and only minor potentiation of dexamethasone-induced killing was observed in MMP7 and MMP8 (Fig. 6e). Importantly however, forskolin potentiated the effect of dexamethasone in 5 of the 10 patient-derived MM cells, and in these cells the combination of dexamethasone and forskolin was always more effective than even a ten times higher dose of dexamethasone alone (Fig. 6e).

As it is well established that BMSC may confer treatment resistance to MM cells^24^, it should be emphasized that forskolin alone or in combination with the conventional anticancer drugs in the present study was able to kill the primary MM cells even in the presence of BMSCs.

## Discussion

Despite the development of new combination therapies during the past 15 years, the majority of multiple myeloma patients relapse, their tumor cells become chemoresistant, and they die from the disease. Furthermore, chemotherapeutic agents used for the treatment of MM have substantial side effects including peripheral neuropathy, thromboembolism, neutropenia, anemia, fatigue and gastrointestinal complications^6^. Hence, there is a need for new treatment strategies aiming at reducing side effects and enhancing the efficiency of existing MM therapies.

We previously showed that single treatment by forskolin kills MM cells *in vitro* and delays tumor growth *in vivo*^8^. Combined with the notion that clinical studies have reported limited side effects of forskolin^20^,^23^, we wished to elucidate the potential of using forskolin in combination with existing therapies for MM. In the present study, we report that combination of forskolin with the common MM therapeutic agents melphalan, cyclophosphamide, doxorubicin, bortezomib, and dexamethasone significantly increased apoptosis compared to single agent treatment. In two different MM cell lines, combination of forskolin with low doses of DNA damaging agents induced the same extent of cell death as two to five times higher doses of the single agent treatment alone. Interestingly, dexamethasone and forskolin had a strong synergistic effect on cell death in H929 and OPM-2 cells. This is in line with a previous report demonstrating synergistic inhibition of MM cells between dexamethasone and PDE inhibitors and/or activators of the G protein coupled receptor adenosine A2A both of which can enhance cAMP levels in the cells^13^,^37^. It should be noted that the combination of forskolin and dexamethasone was not equally potent in all MM cell lines. Hence, despite the fact that the Chou-Talalay method revealed synergistic killing also of RPMI8226 and INA-6 cells, these cells were much less responsive to the combination of forskolin and dexamethasone than were H929 and OPM2-cells.

Forskolin also enhanced melphalan- and dexamethasone-induced cell death in patient-derived primary MM cells. As it is well established that BMSCs may confer treatment resistance to MM cells^24^,^38^,^39^, the ability of forskolin to kill primary myelomas was particularly interesting given the fact that the cells were co-cultured with BMSCs. Importantly, forskolin did not significantly decrease the viability of the BMSCs (data not shown) supporting the perception of low toxicity of forskolin on normal cells. It should be remarked that for four out of the ten MM patients primary cells we tested, forskolin alone had little or no effect on cell death, supporting the notion that forskolin should always be combined with conventional chemotherapy in clinical settings.

In an attempt to elucidate the mechanisms whereby forskolin may interfere with the various MM therapeutic agents, we investigated steps in the apoptotic pathway as well as upstream signaling events. The ability of forskolin alone or in combination with dexamethasone to kill MM cells was confirmed by assessing the cleavage of caspase 3 and PARP. Upstream activation of the intrinsic apoptotic pathway is regulated by BCL-2 family members. In this study, we demonstrated that forskolin plus dexamethasone treatment rapidly increases the levels of the pro-apoptotic BCL-2 member BIM. Notably, knocking down BIM partially prevented the cell death as well the caspase activation induced by the combination of forskolin and dexamethasone, suggesting the involvement of BIM in the apoptotic cell death. The present findings are in accordance with previous studies showing that BIM mRNA and protein expression is increased in response to activation of the cAMP/PKA pathway in certain T lymphoid cell lines^40^,^41^, and that knock down of BIM with shRNA blunts cAMP-promoted apoptosis in the S49 murine T-lymphoma cell line^41^. Microarray analyses have also demonstrated that glucocorticoids can upregulate BIM levels in lymphoid cells^35^,^42^. Hence, BIM has been suggested to be a convergence point for the killing of lymphoid cells by glucocorticoids and treatments that raise the levels of cAMP^40^. Here we show for the first time that forskolin, a natural compound that raises intracellular levels of cAMP, is able to enhance the expression of BIM also in MM cells, and that forskolin synergizes with dexamethasone to induce BIM upregulation and apoptosis in MM cells.

How forskolin upregulates BIM remains unclear. The pro-apoptotic activity of BIM is regulated by transcriptional as well as posttranscriptional mechanisms. The forkhead-like transcription factor FOXO3a is a key transcriptional regulator of BIM^43^, whereas posttranscriptional regulation of BIM occurs through a series of phosphorylation events. ERK phosphorylates FOXO3a thereby increasing its degradation via an MDM2-dependent proteasomal pathway^44^. In addition, ERK has been shown to regulate phosphorylation of BIM_EL_ on serine 69, hence promoting its degradation via the proteasomal pathway^36^. In agreement with these results we found that ERK phosphorylation is inhibited by forskolin, thus providing a possible mechanism whereby forskolin may upregulate BIM.

Interactions between MM cells and the bone marrow microenvironment (BMM) activate a multitude of proliferative and anti-apoptotic signaling pathways, including the Ras–Raf–MAPK kinase (MEK)–extracellular signal-regulated kinase (ERK) pathway and the Janus kinase/signal transducer and activator of transcription (JAK-STAT) pathway^45^. We have previously reported that forskolin kills U266 cells by inhibiting the JAK-STAT pathway^10^, and here we could show that forskolin alone or in combination with dexamethasone, melphalan or bortezomib (Fig. 4 and data not shown) also inhibit ERK-phosphorylation. Combined disruption of ERK-signaling and the JAK-STAT pathway is required to induce apoptosis of MM cells in the presence of BMSCs^46^. Hence, by demonstrating the inhibition of both signaling pathways, the results of the present study support the clinical potential of combining forskolin with conventional therapeutic agents.

Natural compounds such as forskolin are often considered to be affordable and, although not necessarily true, to have low general toxicity^47^. As many natural compounds have the advantage of targeting multiple signaling pathways^48^,^49^,^50^,^51^, a growing number of clinical studies are now evaluating their potentials in cancer treatment in combination with conventional chemotherapies^52^. The present study opens for clinical studies along the same lines. The natural compound forskolin has for centuries been applied in traditional medicine^15^, and its safety has also been documented in conventional modern medicine^23^. We suggest that forskolin, by targeting multiple signaling pathways in MM cells, might reduce the doses of chemotherapeutic agents required to kill such cells *in vivo* and thereby limit the severe side effects associated with current conventional therapies of MM.

## Additional Information

**How to cite this article**: Follin-Arbelet, V. *et al.* The natural compound forskolin synergizes with dexamethasone to induce cell death in myeloma cells via BIM. *Sci. Rep.* **5**, 13001; doi: 10.1038/srep13001 (2015).

## Supplementary Material

Supplementary Information
