# Supplementary material for: The natural compound forskolin synergizes with dexamethasone to induce cell death in myeloma cells via BIM
Source: Sci Rep. 2015 Aug 26;5:13001. doi: 10.1038/srep13001 (PMC4549684; doi:10.1038/srep13001)
Supplement: Supplementary Information [file srep13001-s1.pdf]

The natural compound forskolin synergizes with dexamethasone to induce cell death in myeloma cells via BIM

Virginie Follin-Arbelet, Kristine Misund, Elin Hallan Naderi, Hege Ugland, Anders Sundan, Heidi Kiil Blomhoff

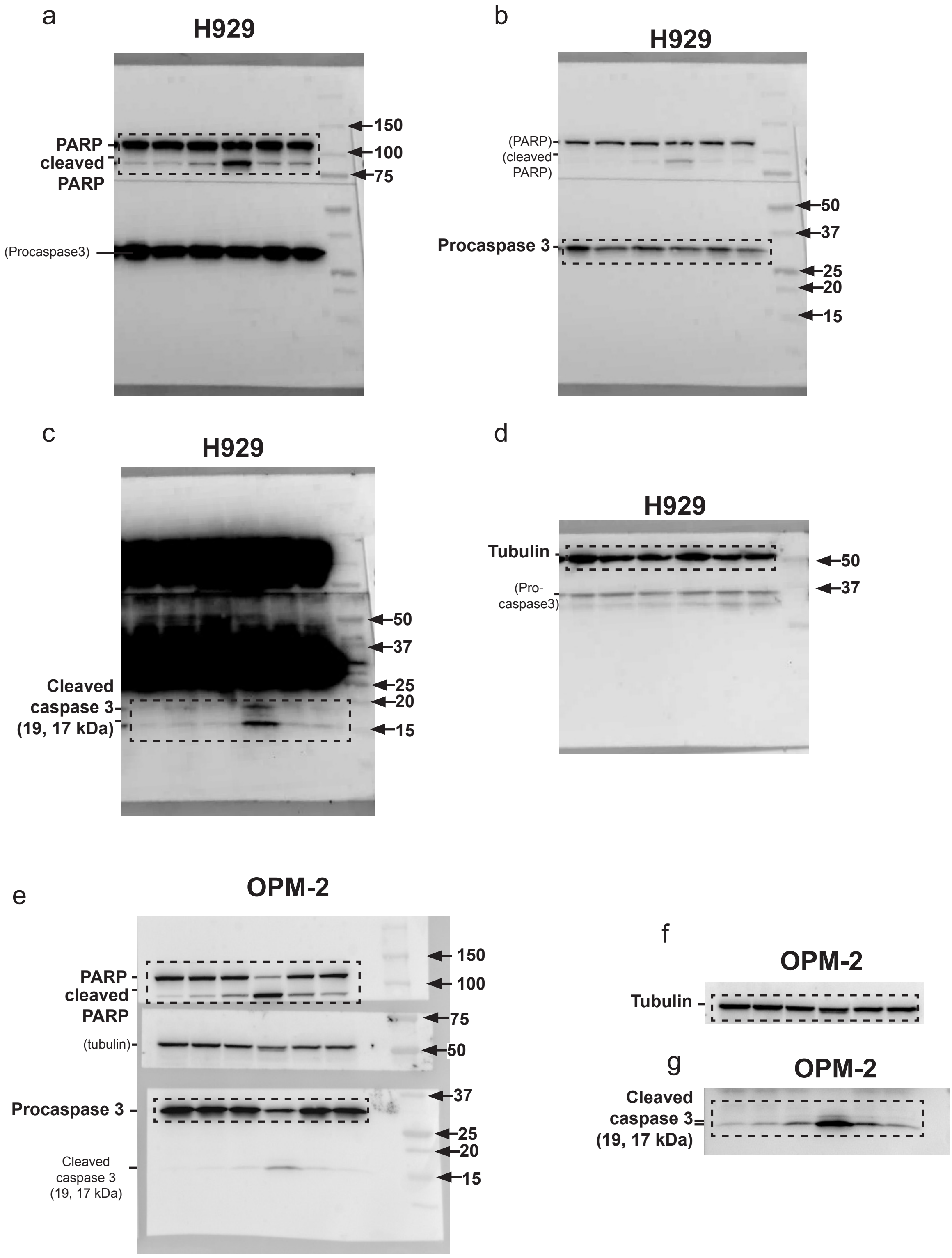

Supplementary Figure S1: Full-length Western blots, which were cropped for Fig. 3d. The dotted lines represent the images shown in the original figures. Panel a, b and c represent multiple exposures of the same blots. The lower blot presented in panel a, b and c was subsequently immunostained to detect tubulin (panel d). Panel e, f and g represent multiple exposures of the same blots.

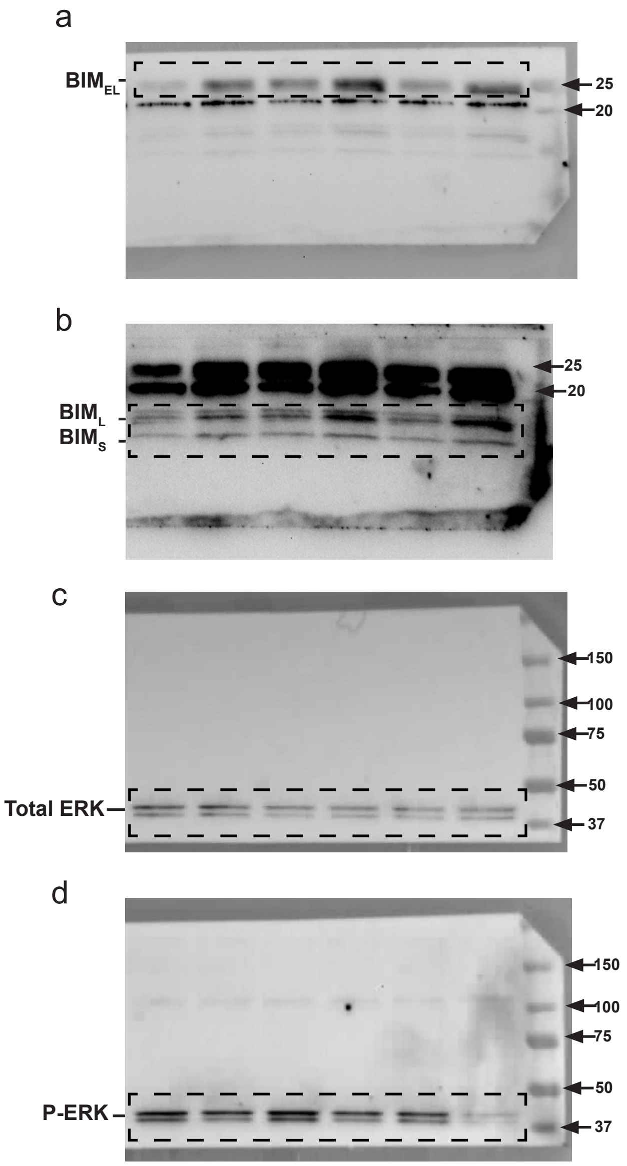

Supplementary Figure S2: Full-length Western blots, which were cropped for Fig. 4. The dotted lines represent the images shown in the original figures. Blot presented in panel a, b and c were run under the same experimental conditions. Panel a and b represent two different exposures of the same blot. After immunosatining with antibody recognizing ERK proteins, the blot from panel c was stripped and immunostained to detect phosphorylated ERK (panel d).

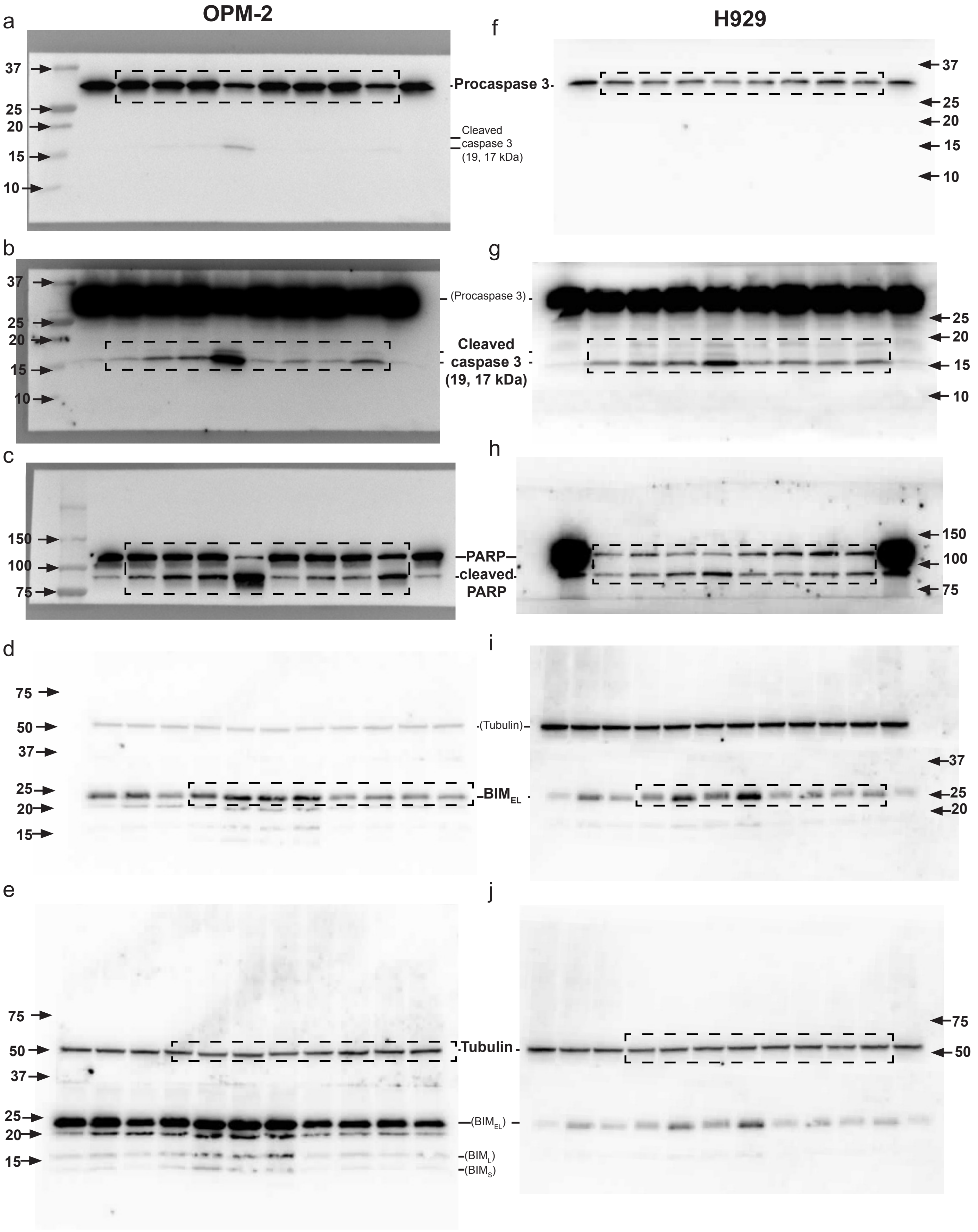

Supplementary Figure S3: Full-length Western blots, which were cropped for Fig. 5a. The dotted lines represent the images shown in the original figures. Blots from panel a, b, c, d and e, and blots from panel f, g, h, i and j were respectively proceeded under the same experimental conditions with the indicated primary antibodies. Panel b, e, g and j represent a higher exposure of blots from panel a, d, f and i, respectively.

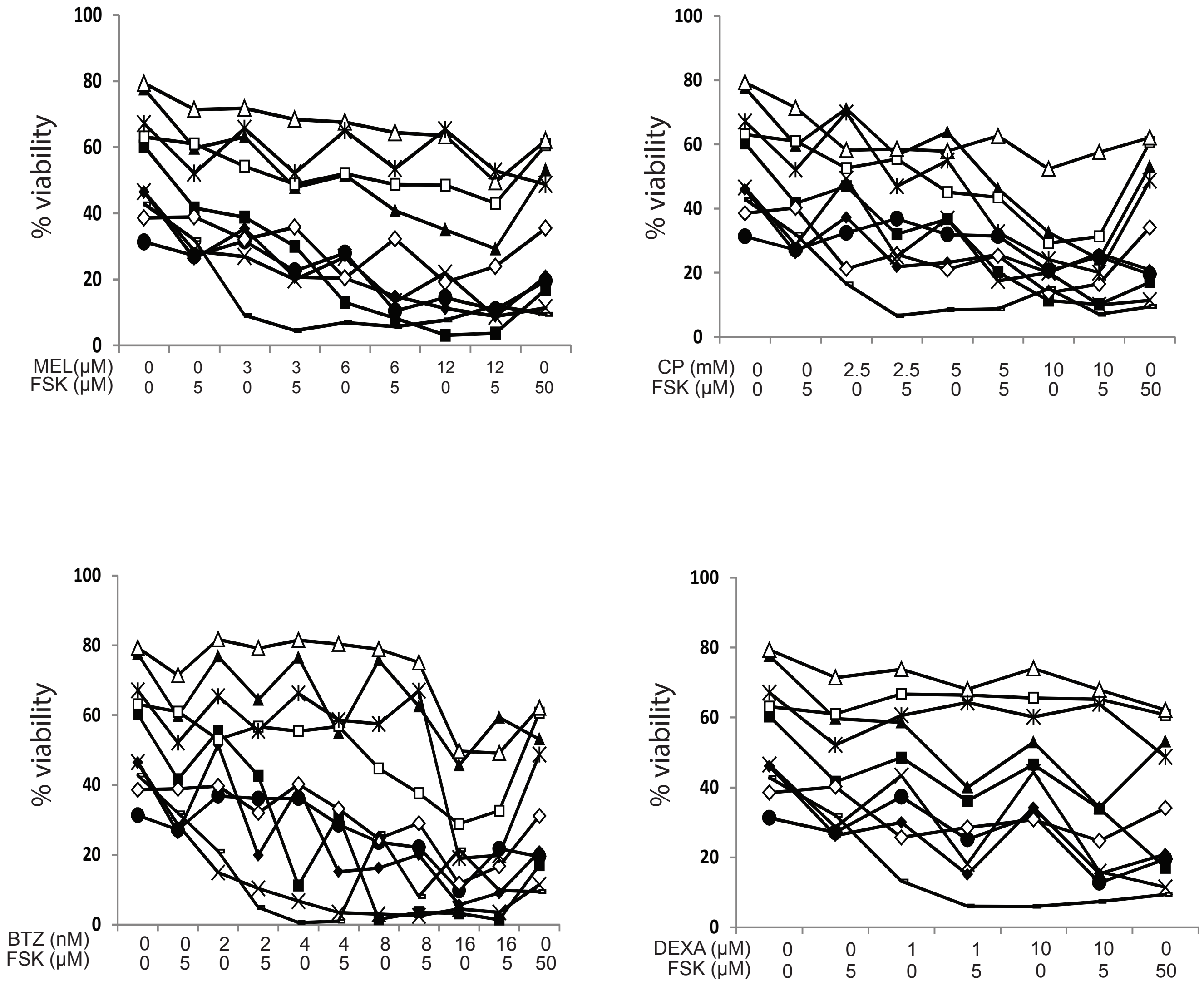

Supplementary Figure S4: Results from Figure 6 presented as line graphs.
